# Stimuli for Adaptations in Muscle Length and the Length Range of Active Force Exertion—A Narrative Review

**DOI:** 10.3389/fphys.2021.742034

**Published:** 2021-10-08

**Authors:** Annika Kruse, Cintia Rivares, Guido Weide, Markus Tilp, Richard T. Jaspers

**Affiliations:** ^1^Department of Biomechanics, Training, and Movement Science, Institute of Human Movement Science, Sport and Health, University of Graz, Graz, Austria; ^2^Laboratory for Myology, Department of Human Movement Sciences, Vrije Universiteit Amsterdam, Amsterdam Movement Sciences, Amsterdam, Netherlands; ^3^Department of Rehabilitation Sciences, Faculty of Kinesiology and Rehabilitation Sciences, University Hospital Leuven, Leuven, Belgium

**Keywords:** growth, muscle-tendon complex, treatment, training, stretching, lengthening, cerebral palsy, hypertrophy

## Abstract

Treatment strategies and training regimens, which induce longitudinal muscle growth and increase the muscles’ length range of active force exertion, are important to improve muscle function and to reduce muscle strain injuries in clinical populations and in athletes with limited muscle extensibility. Animal studies have shown several specific loading strategies resulting in longitudinal muscle fiber growth by addition of sarcomeres in series. Currently, such strategies are also applied to humans in order to induce similar adaptations. However, there is no clear scientific evidence that specific strategies result in longitudinal growth of human muscles. Therefore, the question remains what triggers longitudinal muscle growth in humans. The aim of this review was to identify strategies that induce longitudinal human muscle growth. For this purpose, literature was reviewed and summarized with regard to the following topics: (1) Key determinants of typical muscle length and the length range of active force exertion; (2) Information on typical muscle growth and the effects of mechanical loading on growth and adaptation of muscle and tendinous tissues in healthy animals and humans; (3) The current knowledge and research gaps on the regulation of longitudinal muscle growth; and (4) Potential strategies to induce longitudinal muscle growth. The following potential strategies and important aspects that may positively affect longitudinal muscle growth were deduced: (1) Muscle length at which the loading is performed seems to be decisive, i.e., greater elongations after active or passive mechanical loading at long muscle length are expected; (2) Concentric, isometric and eccentric exercises may induce longitudinal muscle growth by stimulating different muscular adaptations (i.e., increases in fiber cross-sectional area and/or fiber length). Mechanical loading intensity also plays an important role. All three training strategies may increase tendon stiffness, but whether and how these changes may influence muscle growth remains to be elucidated. (3) The approach to combine stretching with activation seems promising (e.g., static stretching and electrical stimulation, loaded inter-set stretching) and warrants further research. Finally, our work shows the need for detailed investigation of the mechanisms of growth of pennate muscles, as those may longitudinally grow by both trophy and addition of sarcomeres in series.

## Introduction

In order to move and perform locomotion, humans and animals generate joint moments by transmitting forces from muscles onto bones. The capability of the muscles to generate force over a range of joint angles is largely determined by optimum muscle length and the length range of active force exertion, which are determined by several morphological characteristics of the muscle. Prime morphological determinants are the number of sarcomeres arranged in series, the physiological cross-sectional area (PCSA, i.e., the cumulative cross-sectional area of all muscle fibers at optimum muscle length) and the pennation angle (PA). The number of sarcomeres in series determines optimum muscle fiber length, which together with the PA determine the muscle belly length as well as the length range over which force can be generated.

The PCSA determines the optimal muscle force and the force generating capacity. The product of optimum muscle fiber length and PCSA is the muscle volume, which contains all sarcomeres arranged in series and in parallel and is indicative of the maximal muscle power generating capacity. Skeletal muscles have a strong ability to adapt during growth and undergo changes in both architecture and contractile properties of their constituents (e.g., O’Brien et al., 2010a,b; Benard et al., 2011; Kubo et al., 2014) and training (e.g., Blazevich et al., 2007; Duclay et al., 2009; Malliaras et al., 2013; Franchi et al., 2014; Noorkõiv et al., 2014; Guex et al., 2016). In general, during development, muscles adapt primarily in response to longitudinal bone growth, and in response to mechanical overload (Toigo and Boutellier, 2006; Schiaffino et al., 2013; Carlson, 2019).

During development, there is the need for skeletal muscles to adapt in length to grow with the longitudinal bone growth (e.g., Haines, 1932). Trophy, i.e., an increase in PCSA, allows for sufficient force exertion over a muscular length range that corresponds to the required joint range of motion during daily life movements (Weide et al., 2015). Since muscle architecture is related to muscle function, limitations in mobility arise in case the ability of a muscle to adapt is restricted, when optimal muscle length is limited such as in athletes with decreased muscle extensibility (Wan et al., 2017) or in case of neuromuscular disorders such as cerebral palsy (CP), respectively (Herskind et al., 2016; Willerslev-Olsen et al., 2018).

Treatment strategies to enhance longitudinal muscle growth and increase both optimal muscle length and the length range of active force exertion are favorable for movement performance of both individuals with neuromuscular disorders and athletes with reduced muscle extensibility. Longitudinal adaptations also improve the muscle power generating capacity, which critically determines movement performance in both populations. Based on animal studies, specific interventions or mechanical loading strategies such as stretching or lengthening contractions are currently applied to induce longitudinal muscle growth. Since these studies have shown that, for instance, prolonged lengthening immobilization results in an increase in serial sarcomere number in animals (Williams and Goldspink, 1978), it is assumed that stretching treatments may also induce such adaptations in humans (e.g., Wiart et al., 2008; Zhao et al., 2011). However, the scientific evidence has not yet confirmed this assumption and the overall effectiveness of stretching in humans is still in question (Katalinic et al., 2011; Novak et al., 2013; Wan et al., 2017). The question remains what triggers longitudinal muscle adaptations during growth and training.

The aim of this review was to identify potential strategies that may induce longitudinal muscle growth in humans. Therefore, we firstly described the key determinants of optimum muscle length and the length range of active force exertion. Secondly, we summarized information on natural growth and the effects of loading on adaptation of muscle and tendinous tissue in healthy animals and humans. Finally, the current knowledge and potential strategies with regard to longitudinal muscle adaptation were discussed, and research gaps were highlighted.

## Determinants of Muscle Length and the Length Range of Active Force Exertion

**Figure 1:**
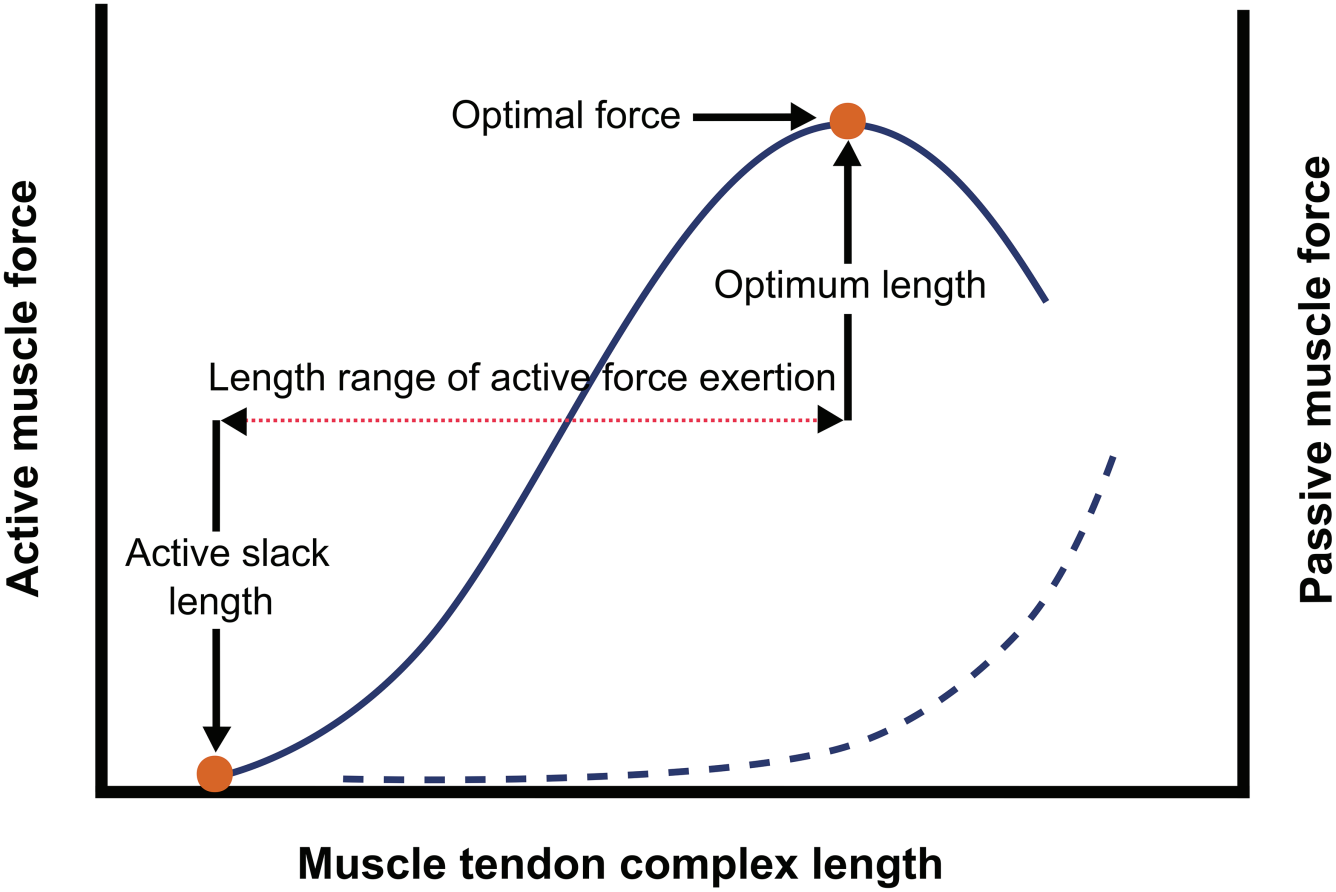
Schematic representation of the active and passive length-force relationship of a muscle. The figure illustrates the active length-force curve (continuous line), the red dots show the active slack length (length at which the muscle does not active exert force) and the optimum muscle length length of the active muscle at optimum length), together with the optimal muscle force, these are measures of the length range of active force exertion (···). The exponential curve (---) shows the passive force, which can be explained as the exerted resistance to stretch caused by passive elastic properties.

The skeletal muscle-tendon complex (MTC) consists of a bundle of striated muscle fibers (i.e., the muscle belly), connected to bones and/or capsules by collagenous structures (i.e., tendon/aponeurosis). Muscle fibers contain sarcomeres (comprised of contractile myofilaments actin and myosin), which are arranged both in parallel and in series. In response to excitation, the muscle belly generates force by contraction of the sarcomeres, pulling origin and insertion of the MTC towards each other. The force exerted actively by a muscle can be expressed as a function of muscle length. The muscle length at which the muscle does not generate actively force while being stimulated is referred to as active slack length. The muscle length at which the muscle exerts maximal active force is referred to as optimum muscle length, the difference between the optimum and the active slack length is referred to as length range of active force exertion (Figure 1). Both the amount of force and the length over which the force is generated are determined by several mechano-morphological muscle properties that are summarized in the subsequent sections.

Skeletal muscles have diffrent types of geometry, parallel fibered muscles and pennate muscles. The contribution of the different determinants of muscle length and length range of active force exertion are largely dependent on the muscle’s geometry. Therefore, within this review, findings are described as much as possible separately for both geometries, without accounting for differences between uni-, bi- and multi-pennate muscles.

### Parallel Arrangement of Sarcomeres

**Figure 2:**
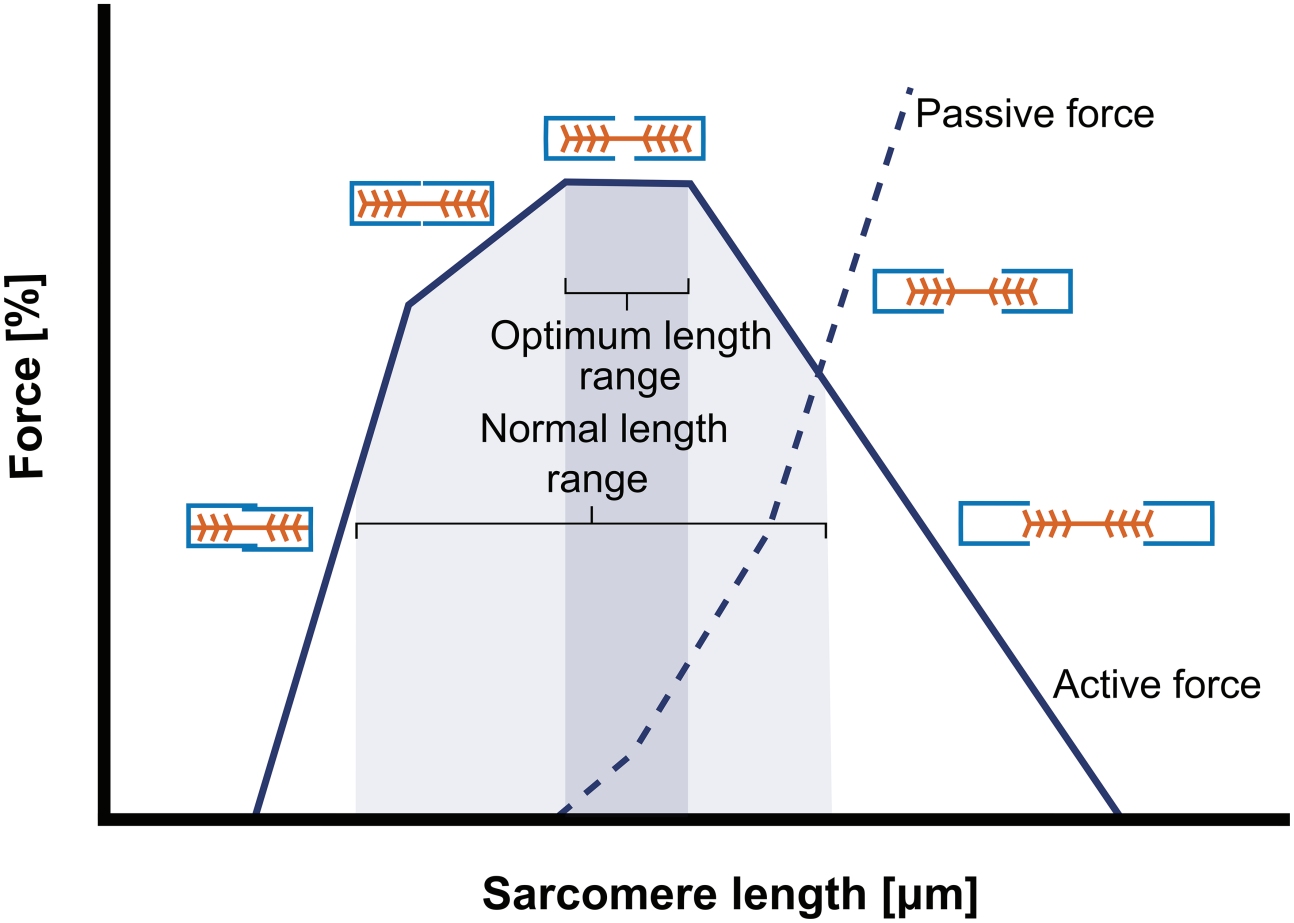
Schematic representation of the sarcomere length-force curve. The figure illustrates the relationship between the amount of actin-myosin overlap and the isometric force generation. At very short sarcomere length force is low “owing to double interdigitation of actin filaments with both myosin and actin filaments from opposite sides of the sarcomere” (Lieber and Ward, 2011). In the ascending limb of the curve, active force (continuous line) of the single sarcomere increases with increasing sarcomeres length. When the sarcomere length is close to optimal (optimal actin-myosin overlap) and active force is maximal, passive force (---) starts to increase. Further increases in sarcomere length results in decreases in actin and myosin overlap, consequently active force decreases and passive force increases. The grey areas in the image represent the optimum length and the normal length range at which sarcomeres are usually functioning, both differ between species and muscles.

The muscle cross-sectional area perpendicular to the muscle fiber axis, which is determined to the number of muscle fibers, is called PCSA. Optimal muscle force is proportional to the number of muscle fibers that are recruited by stimulation, the number of parallel arranged sarcomeres within the muscle fiber (i.e., fiber cross-sectional area (fCSA also ‘fiber diameter’)), the amount of overlap of actin and myosin (Huxley and Hanson, 1954; Figure 2), as well as the stimulation frequency.

**Figure 3:**
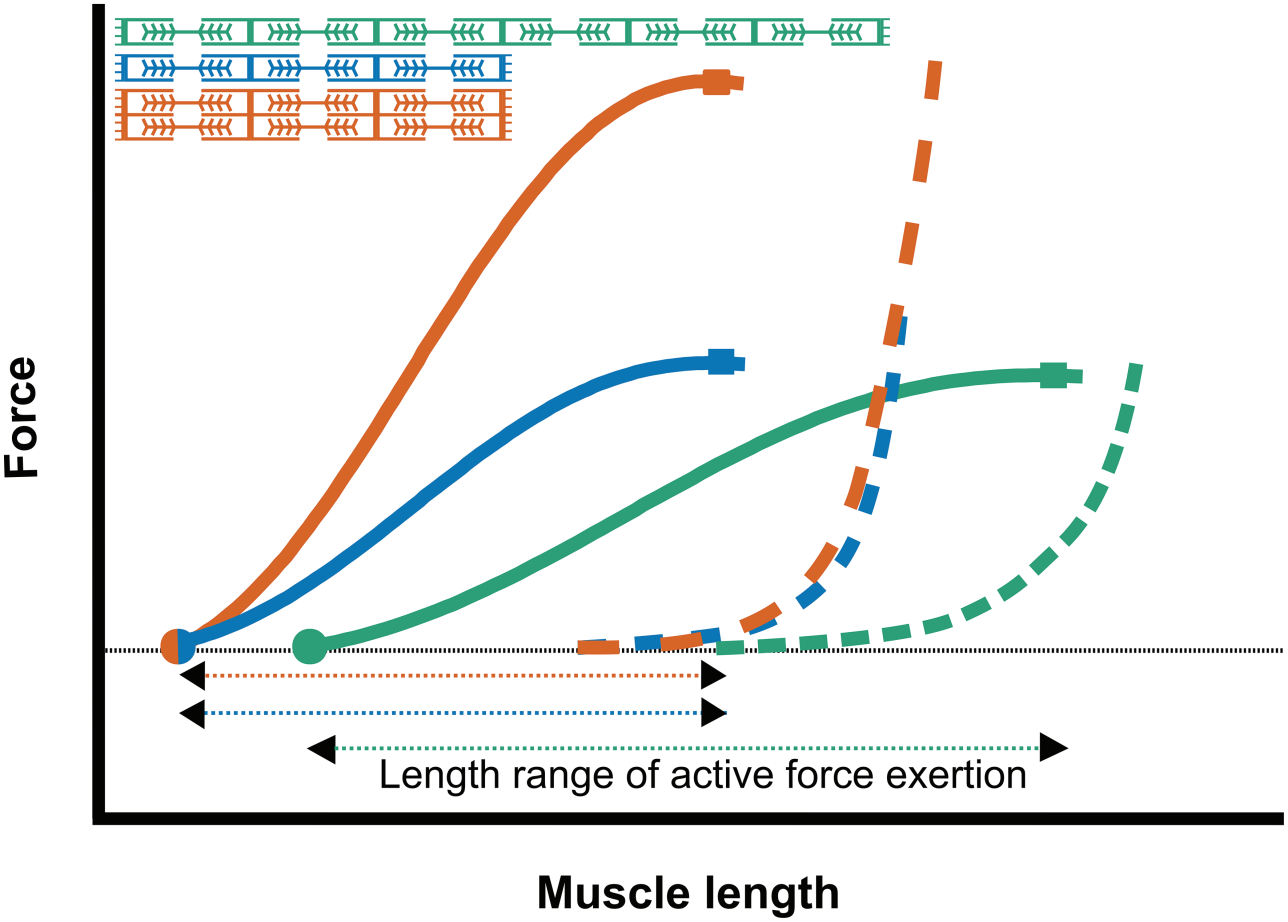
Schematic representation of the length-force relationship of muscles with different sarcomere numbers The active and passive force relationship of a muscle fiber with a low number of parallel and serial sarcomeres (blue); a muscle fiber with double number of parallel sarcomeres (red); and a muscle fiber with double number of serial sarcomeres (green). Increasing the number of sarcomeres in series results in increased active slack (dots) and optimum length (squares), as well as an increase in the length range of active force exertion. Increasing the number of parallel sarcomeres results in a higher optimal muscle force, without increasing the active slack and optimum muscle length or the length range of active force exertion.

The number and length of the cytoskeleton proteins, and the amount of connective tissue arranged in parallel determine the passive force over the muscles’ length range of active force exertion (Wang et al., 1991; Figure 3). Therefore, passive muscle force is positively related to the PCSA.

**Figure 4:**
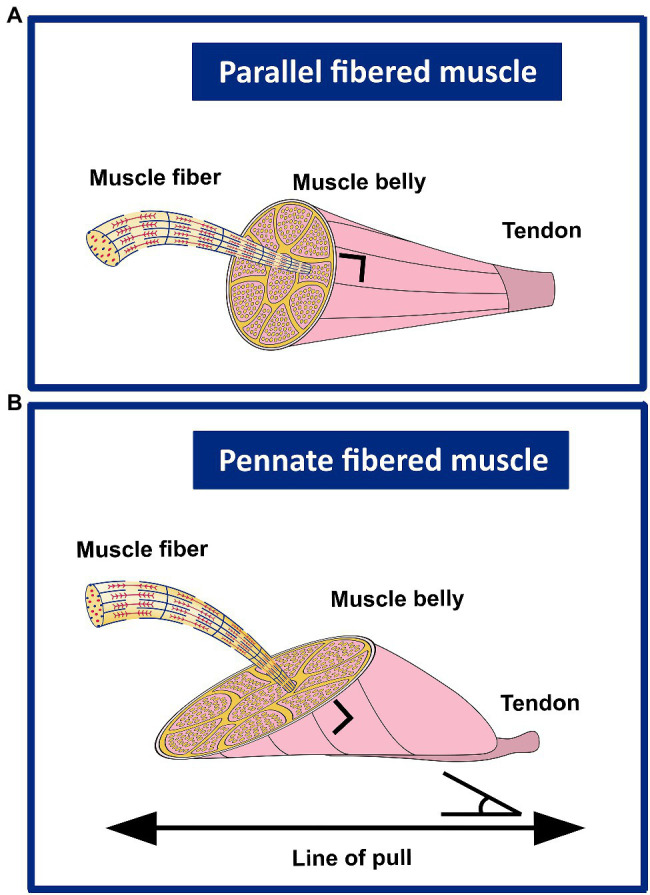
Schematic representation of the contribution of sarcomeres (serial and in parallel) to the muscle belly length, as well as PCSA in parallel and uni-pennate fibered muscles. **(A)** In parallel fibered muscles, the length of the muscle belly is determined by the number of sarcomeres in series, as well as the slack, optimum length and the length range of active force exertion, whereas sarcomeres arranged in parallel determine the (fiber) CSA. **(B)** In pennate fibered muscles, the muscle belly length is determined both by sarcomeres in series, as well as by the number of sarcomeres in parallel. The number of sarcomeres in parallel also determines the (fiber) CSA. This latter is described as the area of the transversal section perpendicular to the muscle fiber direction. In pennate muscles, the PCSA contributes to the optimum length and range of active force exertion. When muscle fibers that are under a line of pull generate force, the contribution to the muscle length is less than that of the muscle fiber length itself.

Figure 4 shows a schematic representation of the contribution of sarcomeres to the morphology in parallel and pennate fibered muscles.

### Serial Arrangement of Sarcomeres

Optimum muscle length, active slack length and the muscle length range of active force exertion are largely determined by optimal sarcomere length and the number of sarcomeres arranged in series (Huxley and Hanson, 1954; Figure 3). Moreover, the number of sarcomeres in series is strongly correlated with the maximal contraction velocity of muscles (Hill, 1938; Wilkie, 1949; Wickiewicz et al., 1984; Lieber and Friden, 2000). In addition, serial distribution of sarcomere lengths within muscle fibers and distribution of fiber mean sarcomeres lengths between muscle fibers are associated with a reduced optimal force and an enhanced muscle length range of active force exertion (Huxley and Peachey, 1961; Lieber and Baskin, 1983; Willems and Huijing, 1994). The relation between fiber mean sarcomere length distribution (i.e., heterogeneous lengths) and muscle length range can be explained by the length-force relationship of individual sarcomeres and fibers constituting the muscle. In case of heterogeneous lengths, some sarcomeres within a fiber reach their optimum length, or a length over optimum, while other sarcomeres will still have a length on the ascending limb of the length-force relation. In comparison to a muscle consisting of homogeneous fiber mean sarcomere lengths, a muscle with heterogeneous lengths has a lower optimal force and a wider length range of active force exertion.

### Pennation Angle

In parallel fibered muscles, optimum muscle length and muscle length range of force exertion are predominantly determined by optimum muscle fiber length and the muscle fiber length change (active slack length to optimum length). However, most human skeletal leg muscles are pennate (Ward et al., 2009). The pennation angle (PA) describes the angle of the muscle fibers with respect to the line of pull of the muscle. In pennate muscles, the optimum muscle length is negatively related to the PA and the length range at which force can actively be exerted is positively related to the PA. To be more specific, a high pennate muscle implies a shorter optimum muscle length and a greater length range over which force can be actively exerted. Moreover, in muscles with a certain degree of pennation, muscle length can increase by muscle fiber hypertrophy while the muscle fiber length may remain unaltered. Furthermore, the PA is negatively related to the muscle length, it is considerably greater at active slack length compared to optimum muscle length (Zuurbier and Huijing, 1993).

### Intramuscular and Tendinous Connective Tissue

Intramuscular connective tissue (including the aponeurosis, which is an intramuscular tendon) and tendons are responsible for force transmission from sarcomeres to bones (Huijing, 2009). Force generated by sarcomeres is transmitted to connective tissue at the myotendinous junction, and laterally to connective tissue structures surrounding the muscle fibers, fascicles, and subsequently to the tendons. The collagen composition and cross-linking of connective tissue determines the mechanical properties of these structures and influence the muscle active, as well as passive length-force characteristics.

Due to their elastic properties, tendons contribute to the extensibility of the MTC. The degree of elongation depends on the force exerted and the stiffness of the tendon. Both active and passive MTC length-force characteristics are affected by the length and elastic modulus of the tendon. The aponeurosis length of an active muscle is determined by its thickness and material properties and depends on muscle length, muscle fiber force, and degree of activation (Ettema and Huijing, 1989; Ettema et al., 1990; Zuurbier and Huijing, 1993; Scott and Loeb, 1995; Raiteri, 2018).

The collagenous structures surrounding the muscle and muscle fibers influence the active and passive muscle force transmission, however, are beyond the scope of this review (for details: Zuurbier et al., 1994 or Huijing, 2009).

Other determinants of muscle geometry are the curvature and 3D orientation of muscle fibers. Muscle fibers located at the outside of the muscle are more curved than internally located muscle fibers (Otten, 1988). Muscle fibers located more towards the proximal and distal ends of the muscle bulge because the cumulative attachment area of the muscle fibers at the aponeurosis does not accommodate to the increase in muscle fiber diameter when the muscle shortens (Otten, 1988; Huijing, 1998). Due to the bulging, the angles of the muscle fibers with the respective aponeuroses are at one end larger and at the other end smaller than straight fibers and as such have only limited effect on the muscle optimal force. When muscle length increases, the curvature of muscle fiber decreases due to the decrease in muscle fiber cross-sectional area. Note that at both lateral sides of the mid-longitudinal plane of a pennate muscle, muscle fibers are also slightly oriented in a lateral direction, reducing their forces exerted in the direction of the muscle line of pull. The magnitudes of the effects of curvature and 3D orientation on the length-force characteristics are not well known. Using 2D linearized muscle geometry modelling of rat GM, based on the dimensions and geometry of the muscle mid-longitudinal plane and neglecting curvature and variations in 3D orientation, yielded rather similar length-force characteristics as those experimentally assessed (slack and optimum length are less than 2% different; c.f. van der Linden et al., 1998). These data suggest that for rat GM effects of bulging seem relatively small compared to those of other morphological variables such as muscle fiber length, PCSA and PA (van der Linden et al., 1998).

## Animal Experimentation

### Typical Muscle Growth in Animals

During development, bone growth causes tensile stress on skeletal muscles and increases muscle excursion, both are presumed to stimulate (Haines, 1932; Crawford, 1950) longitudinal muscle growth, whereas increased body mass is highly related to the increase in muscle PCSA (Woittiez et al., 1986; de Koning et al., 1987; Papenkort et al., 2020). Muscle growth can be modulated by alterations in external stimuli applied to (e.g., immobilization and inactivity, physical activity, mechanical loading, hormones, nutrition). The morphological changes during muscle growth and adaptation in response to changes in physiological conditions are discussed in the following paragraph.

In both parallel fibered and pennate muscles of both rats and rabbits, optimum muscle belly length (Alder et al., 1958; Woittiez et al., 1986; de Koning et al., 1987; Willems and Huijing, 1992) and the muscle length range of active force exertion (Woittiez et al., 1989) increase during development. Muscle fibers grow longitudinal by addition of sarcomeres in series (Williams and Goldspink, 1971; Tardieu et al., 1977). The rate of longitudinal muscle fiber growth in rodents is fast during the first four weeks after birth and then levels off. In addition, the rate of longitudinal muscle fascicle growth within lower limb muscles is muscle specific. For some muscles (i.e., gastrocnemius lateralis (GL) and GM, as well as flexor digitorum longus in rabbits), muscle fascicle length reaches a maximal value after 6weeks after birth, while other muscles ((m. tibialis anterior and m. plantaris) in rabbits continue increasing their length at a constant rate) until at least 15weeks after birth (Böl et al., 2017; Siebert et al., 2017). In rat parallel fibered muscles, growth is only determined by longitudinal muscle fiber growth (Willems and Huijing, 1992), while in pennate muscles of rats and ducks, longitudinal muscle growth also occurs by radial growth of muscle fibers (Swatland, 1980; Woittiez et al., 1986; Papenkort et al., 2020).

During development, muscle PCSA increases (Woittiez et al., 1986, 1989; de Koning et al., 1987; Papenkort et al., 2020, 2021) by an increase in fCSA (Swatland, 1980; Alnaqeeb and Goldspink, 1987; Willems and Huijing, 1992). Although some studies (e.g., Enesco and Puddy, 1964; Ross et al., 1987) have reported hyperplasia during postnatal development in rat muscles, more have shown that hyperplasia does not occur in muscles from rodents (Goldspink, 1972; Timson and Dudenhoeffer, 1990; White et al., 2010). Therefore, the fiber number does not account for increases in PCSA during postnatal development.

Due to longitudinal muscle fiber growth and fiber hypertrophy during development, the muscle will function at an absolute higher muscle length, attaining a higher slack and optimum length, as well as a higher length range of active force exertion (Woittiez et al., 1986; de Koning et al., 1987).

In an almost completely parallel fibered muscle (i.e., semimembranosus lateralis of the rat), the fiber PA has been found to increase during development (Willems and Huijing, 1992). In pennate muscles of growing rats and rabbits (GM) and rabbits (soleus and plantaris muscles), it seems that the PA increases only slightly during growth (Heslinga and Huijing, 1990; Papenkort et al., 2020, 2021). Note that changes in PA over age may differ between muscle types (*cf.* Siebert et al., 2017).

In rodents, longitudinal bone growth continues beyond young-adulthood (Heslinga and Huijing, 1990). In mature rats, muscle morphology seems to adapt in proportion to longitudinal bone growth. However, these morphological adaptations differ from those at young age (Heslinga and Huijing, 1990; Heslinga et al., 1995). In mature rats, increases in muscle mass, PCSA, and serial sarcomere numbers could only be shown in the parallel fibered soleus muscle (i.e., pennation angle <4 degrees). In contrast, in pennate muscles, increases in muscle length are attained by increases in the length component of the PCSA, aponeurosis length, and aponeurosis angle (Heslinga and Huijing, 1990). Such results indicate that during adulthood, adaptation of pennate muscle of rodents seems to differ from changes in mechanical demand than during childhood or adolescence.

#### Effect of Growth on Aponeurosis and Tendon Dimensions

During growth, the increase in muscle PCSA in rats and rabbits is accompanied by an increase in aponeurosis length and width (Woittiez et al., 1989; Willems and Huijing, 1992; Böl et al., 2017; Siebert et al., 2017), accommodating the greater fCSA. Tendons of rats and rabbits also increase in length and thickness (Alder et al., 1958; de Koning et al., 1987; Woittiez et al., 1989; Böl et al., 2017; Siebert et al., 2017). The increase in tendon CSA occurs by the increase in CSA of tendon fibers (Woittiez et al., 1989; Nakagawa et al., 1994; Nakagawa, 1996). The longitudinal and radial tendon growth affects their mechanical properties, such as tensile strength, and stiffness (Haut and Little, 1972; Walker et al., 1976; Woo et al., 1980; Shadwick, 1990; Nakagawa, 1996). However, tendon and muscle belly do not grow proportionally. The growth rate of tendons and aponeuroses are muscle-specific (Böl et al., 2017; Siebert et al., 2017). As the growth ratio differs considerable between muscles, even from the same species, the tendon-muscle belly or fascicle length ratio differ between muscles and are dependent on the growth stage (Böl et al., 2017; Siebert et al., 2017). For example, the GM and GL of rabbits showed high tendon-muscle fascicle length ratios 3weeks after birth, the ratio had almost doubled by the age of 14weeks (Siebert et al., 2017). This was explained by a lack of longitudinal muscle fascicle growth and a large increase in aponeurosis, as well as tendon length during growth. In contrast, for tibialis anterior muscle of rabbits at the age of 3weeks, a low tendon-muscle fascicle length ratio was shown, which was slightly decreased by the age of 14weeks (Siebert et al., 2017). The tibialis anterior showed a linear increase of the fascicle lengths during growth, while the growth ratio of the tendon and aponeurosis were low.

### Adaptive Responses to Increased Contractile Activity and Mechanical Loading

The effects of alterations in the magnitude of contractile activity on muscle morphology and function have been extensively studied over the years in animal models. These models have been used to study the effects of prolonged muscle activity, mechanical overload, stretching, and combinations of these. Different approaches and devices (e.g., treadmill running and electrical muscle stimulation) have been used to study the effect of physical activity in animals. Mechanical loading was induced by several different overloading models, such as synergist ablation and denervation, unloading of the front paws or hind limbs, or by using weights. Animal studies with prolonged activity models simulate an increase in muscle endurance and/or in repetitions, whereas increased mechanical load simulates an increase in strength. Muscle immobilization and stretching were further used to study its effects on muscle length, the range of active force exertion and muscle stiffness.

The “active position hypothesis” (i.e., sarcomere lengths at which muscles are mostly active) by Herring et al. (1984), states that the number of sarcomeres in series is regulated to assure that each single sarcomere is at the optimum length on its active length-force curve, which is near to the joint position where the highest force is exerted and motor units of the muscle are fully recruited.

#### Effects of Overload on Muscle Growth

Mechanical loading of skeletal muscle may occur by (active) contraction at high resistance or by applying external tensile forces onto the muscle. Different models cause high mechanical loading and stimulate skeletal muscle hypertrophy, for example, weightlifting and synergist denervation. In addition, mechanical loading of muscle may also originate from surrounding muscle tissue *via* myofascial force transmission (Huijing, 2009). Muscle fiber hypertrophy in the flexor carpi radialis muscle has been reported in cats after a period of weight lifting which was accompanied by an increase in power generating capacity (Gonyea, 1980). Increased load, due to resection of synergistic muscles (soleus and GM) showed that muscle fibers from the plantaris muscle increased their CSA (Vaughan and Goldspink, 1979). Van der Meer et al. (2011) showed that denervation of the plantaris synergists muscles caused a considerable addition of myonuclei, which was followed by hypertrophy in the plantaris of young-adult and old rats. As a result of increased CSA, caused by increased mechanical loading, passive force increases as well, and thus affects the length-force relationship of the muscle.

#### Effects of Prolonged Muscle Activity on Muscle Growth

The effects of activity on muscle length seem to be highly dependent on the length at which the muscle is maintained or the length range over which the muscle is operating. Low frequency stimulation of the low pennate soleus muscles of rabbits (i.e., 10–13 degrees PA (Siebert et al., 2017; Papenkort et al., 2020)) showed increases in their number of sarcomeres in series only when the muscles were kept at high length, the opposite occurred at short length (Williams et al., 1986). The reduction of the serial sarcomere number was higher than that of muscles immobilized at short muscle length without activation. These findings indicate that the serial sarcomere adaptation is enhanced by contractile activity. Moreover, activation seems to prevent connective tissue accumulation caused by immobilization (Williams et al., 1988).

In addition to morphological changes, activation seems to affect muscle phenotype. For instance, low-frequency muscle stimulation was reported to stimulate the adaptation towards a slow-contracting muscle (i.e., higher amount of slow muscle fibers; Pette and Vrbová, 1999). This should be considered when applying as a treatment since these fibers have a lower power output and smaller fCSA. Specific consequences of this change in phenotype, for muscle force in parallel fibered muscles and muscle length in pennate muscles are yet not known.

In summary, based on the data of different animal models, it is concluded that mechanical overloading of muscle leads to muscle fiber hypertrophy (i.e., increase of the fCSA and PCSA) in both parallel fibered and pennate fibered muscles. However, longitudinal muscle growth, due to increased muscle activity has only been shown in parallel fibered muscles when placed in a lengthened position. Moreover, the activity as a sole intervention regulates the addition and subtraction of sarcomeres in series depending on the muscle length during muscle activity.

#### Muscle Adaptations in Response to Concentric and Eccentric Exercise Interventions

To the best of our knowledge, there are no studies showing the effects of isometric contraction training on muscle function or morphology of animal muscles. Therefore, only the effects of concentric and eccentric exercise interventions on muscle growth of animal muscles are summarized in the following sections.

Concentric contractions are characterized by muscle shortening while force is actively produced. Uphill walking that is characterized by concentric activity at short muscle length, and promotes the loss of serial sarcomeres in the vastus lateralis (VL) and intermedius of rats (Butterfield et al., 2005). Furthermore, Lynn et al. (1998) showed that the vastus intermedius of rats running uphill had less sarcomeres in series than that of rats running downhill. Moreover, the range of the torque-angle curves of the animals performing concentric exercise was smaller than that of animals which performed eccentric exercise.

Although these studies reported slightly different results, it seems that animal hind limb muscles reduce their number of sarcomeres in series within 5 to 10days after starting concentric exercises at short muscle length.

Eccentric contractions are characterized by MTC lengthening while muscles are actively exerting a resistance to lengthening. Effects of eccentric contractions are determined by the uniformity of the muscle architecture and the length range over which the muscle is eccentrically contracted.

Eccentric exercise at short muscle length was shown to cause only a regional addition of sarcomeres in series in the tibialis anterior (TA), but no addition in the extensor digitorum longus of rabbits (Koh and Herzog, 1998; Butterfield and Herzog, 2006). Moreover, downhill running with a 20° decline did not increase the number of sarcomeres in series in the soleus and extensor digitorum longus in rats (Chen et al., 2020). Neither did downhill running with a 14° decline in the GM, vastus medius and lateralis in mice (Morais et al., 2020). In contrast, the vastus intermedius (low PA) of rats who ran downhill with a 16° decline on a treadmill showed a substantial increased number of sarcomeres in series compared to sedentary rats and rats that ran uphill (i.e., concentric contractions; Lynn and Morgan, 1994). Similar results were found by Butterfield et al. (2005) in the vastus intermedius and lateralis. These results suggest that a non-uniform muscle architecture causes the lack of adaptation to eccentric exercise, as the non-uniformity results in differences in local strains.

Furthermore, eccentric exercises performed at high muscle length of the TA of rabbits did not affect the number of serial sarcomeres in the lateral muscle regions, which was similar to control muscles (Butterfield and Herzog, 2006). Whereas in the medial, central superficial, and deep regions, the number of serial sarcomeres increased.

Eccentric contractions performed over the full muscle length range of the TA resulted in an increased number of sarcomeres, whereas contractions performed over a small muscle length range reduced the number of sarcomeres in series compared to control muscles (Butterfield and Herzog, 2006).

Eccentric exercises also stimulate the addition of parallel arranged sarcomeres within the muscle fibers resulting in increases in both fCSA and the PCSA, moreover, when applied to the muscle at long lengths showed higher increases in PCSA than those performed at smaller muscle lengths (Butterfield and Herzog, 2006). Eccentric exercises improve the length force-characteristics by improving both the maximum force and length range of active force exertion.

### Immobilization

Joint immobilization is a common intervention to maintain a muscle at a certain length (e.g., in a shortened, neutral or lengthened position) by fixating the joint(s) over which a target muscle generates joint moments. In this review, we describe the effects of lengthened immobilization (e.g., target muscles are lengthened by moving joints in full plantar or dorsi flexion) on muscle length and the range of active force exertion.

#### Effect of Immobilization on Muscle Force and Length of Parallel Fibred Muscles

As in muscles with a low degree of pennation (i.e., parallel fibered), changes in optimum muscle length are only achieved by changes in the number of sarcomeres in series. Immobilization in a lengthened position (i.e., full dorsi flexion), results in a considerable addition of serial sarcomeres (Tabary et al., 1972; Williams and Goldspink, 1973; Williams and Goldspink, 1978; Soares et al., 2007), ensuring that the optimum muscle length is attained in the imposed immobilized joint position.

Lengthening immobilization of mouse soleus muscle was shown to induce an increase of the optimal muscle force and a shift of the optimum length to longer muscle lengths. Moreover, immobilized and control muscles showed similar passive length-force curves (Williams and Goldspink, 1978). In addition, lengthening immobilization of the soleus, GM and tibialis anterior muscles in rats leads to hypertrophy (Coutinho et al., 2004). Lengthening immobilization of the rabbit extensor digitorum, tibialis anterior and soleus muscles stimulates the IGF-1 expression within these muscles. IGF-1 is an anabolic growth factor, stimulating muscle hypertrophy by enhancing the rate of protein synthesis and inhibiting protein degradation (Yang et al., 1997; Glass, 2005). These results suggest that lengthening immobilization increases the PCSA of immobilized muscles.

#### Effects of Immobilization on Muscle Force and Length of Pennate Fibred Muscles

At young age, in pennate rat muscles, optimum length is increased by both an increase in the number of sarcomeres in series and PCSA (Heslinga and Huijing, 1993), while in young adult rats the increase in optimum length is mainly due to an increase in PCSA (Heslinga et al., 1995). However, the effects of long length immobilization in those muscles have not been widely studied in animals. Though, there is evidence that pennate muscles immobilized in a lengthened position add sarcomeres in series and reduce their sarcomere lengths (Heslinga et al., 1995).

#### Effects of Continuous High or Low Strain on Single Muscle Fiber Size Adaptation

Culture of single fiber (iliofibularis muscle of *Xenopus laevis)* at low strain, comparable with short muscle length immobilization *in vivo*, did not show a reduced number of sarcomeres in series or atrophy. Immobilization at high strain did not show the expected trophic response (in neither longitudinal nor radial direction; Jaspers et al., 2004). The lack of the expected trophic adaptation suggests that either mechanical interaction with neighboring (muscles)-fibers is needed to trigger adaptation of muscle fiber size (Jaspers et al., 2006) or other factors *in vivo* (e.g., growth factor or cytokines) differ *in vivo* from that *in vitro*.

#### Young Muscle and Tendon Adaptation to Immobilization

Immobilization in a lengthened muscle position in young (1week of age mice; 4weeks old rabbit) muscles resulted in a lack of increase in sarcomeres in series, also the length of the sarcomeres remained unchanged (Tardieu et al., 1977; Williams and Goldspink, 1978). Moreover, tendon length increased such that the mechanical stimuli (strain and force) on the sarcomeres remained low (Tardieu et al., 1977; Williams and Goldspink, 1978; Blanchard et al., 1985).

#### Effects of Immobilization Combined With Enhanced Contractile Activity

Studies investigating the effects of rabbit muscle immobilization at an extended length in combination with electrical stimulation showed increases in muscle length by an addition of serial sarcomeres after only 4days when compared to non-stimulated control muscles or to muscles which were only immobilized muscles (Cotter and Phillips, 1985; Williams et al., 1986). Immobilized soleus muscles of cats in extended position increased their number of sarcomeres in series, irrespectively whether the muscle was innervated or not, however, the rate of increase in number of sarcomeres in series was lower in denervated muscle (Goldspink et al., 1974). These results suggest that neural drive is not necessary for longitudinal muscle adaptation and that the number of sarcomeres in series may be regulated predominantly by passive force.

Furthermore, young denervated cat soleus muscle (pennate muscles) immobilized in an extended position had serial sarcomere addition and less tendon length growth than in innervated muscles (Blanchard et al., 1985). Treatments aiming to lengthen muscles or to increase the range of force exertion in children with neuromuscular disorders may benefit from such effects.

### Effects of Stretching

#### Effects of Passive Stretching on Muscle Growth

Stretching is often defined as a form of physical exercise that includes several sets of stretching stimuli applied to target muscles during a relative short amount of time to target muscles. Static passive stretching has been found to slightly increase the number of sarcomeres and the fCSA in a fully extended soleus muscle of Wistar rats (Coutinho et al., 2004). A study with incremental static stretching of the latissimus dorsi of rabbits during 3 weeks (5% above the optimum length every week), resulted in an increase in serial sarcomere number, muscle mass, as well as fCSA of type 1 fibers (Cox et al., 2000). The collagen content, on muscular level, was also increased, however, returned to baseline values when the stretch was maintained for 6weeks. The tetanus force and the maximal muscle power decreased. This latter might be explained by the shift in MHC muscle fiber composition from fast fatigable towards more slow twitch fibers. Moreover, the fCSA of type I fibers increased by 30%, and the fCSA in type 2B fibers decreased slightly. The adaptations on collagen content are opposite to those of muscles subjected to electrical stimulation (Williams et al., 1988), and may emphasize the importance of activity to prevent an increase of collagen within the muscle.

#### Effects of Intermittent Static Stretching on Muscle Growth

Intermittent stretching is characterized by holding a muscle under stretch for a certain period interrupted by short breaks in which no force is applied. Passive intermittent static stretching of the soleus muscle of rabbits increased the number of sarcomeres in series and muscle mass (de Jaeger et al., 2015). No changes were observed in the number of sarcomeres or muscle mass of the gastrocnemius and plantaris muscles. The passive torque- ankle angle relationship and muscle stiffness remained unchanged. Moreover, intermittent stretching prevents the loss of sarcomeres in series and loss of range of force exertion in muscles immobilized in a shortened position (Williams, 1990). These results suggest that the effects of static stretching are dependent of the muscle architecture.

#### Effects of Passive Stretching Combined With Enhanced Contractile Activity on Muscle Growth

To date, no animal studies are known that investigated the effects of passive stretching combined with enhanced contractile activity on serial sarcomere addition. However, to the best of our knowledge, there is only one study that assessed the effects of stretching combined with enhanced contractile activity on muscle PCSA (Stauber et al., 1994). This study showed that muscle hypertrophy was dependent on stretch velocity. Slow stretching of soleus rat muscles (10mm/s) resulted in increased muscle mass and mean fCSA. In contrast, fast stretching (25mm/s) of the soleus induced an increase in muscle mass, while fCSA was reduced and connective tissue content was increased. The reduced muscle fiber size in combination with the increase in connective tissue suggests that fast stretching causes muscle fiber injury, an increase in variation in muscle fiber diameter, and concomitant fibrosis. These results indicate that slow velocity stretching increases muscle size and likely improves muscle force generating capacity, whereas fast stretching is not beneficial for muscle integrity and force generating capacity. Further studies are required to investigate the underlying mechanisms of muscle fiber injury caused by fast stretching, and the possible implications for human muscles.

## Human Studies

### Typical Muscle Growth in Children and Adolescents

In contrast to studies concerning growth-related muscle and tendon changes in animals, studies in humans are scarce and the existing knowledge mainly comes from cross-sectional studies. These studies may give a certain overview of occurring changes, however, longitudinal studies are still needed to understand critical phases in tissue development during growth (Table 1). Since biological maturation may vary in level, timing, and rate between individuals of the same chronological age (Mirwald et al., 2002; Lloyd et al., 2014), future studies should also evaluate the maturity status of the subjects.

**Table 1:** Summary of the identified research gaps and deduced ideas for future studies on muscle-tendon properties in animal and human research.

Studies	Identified research gaps	Ideas/aspects for future studies
Animals		
Immobilization	Studies are missing that examined effects of immobilization (at long muscle length) in pennate fibered muscles Lack of studies reporting regional effects of immobilization No studies are known that examined the effects of immobilization (at long muscle length) on muscles with contractures (with clinically short length)	Focus on the effects of long length immobilization on morphological adaptation and changes in muscle function Focus on regional adaptation and the effects of these to the muscle length, length range and function Focus on lengthening (e.g., by addition of sarcomeres (both in serial and/or in parallel)
Loading and stretching	Studies are missing that assessed effects of training on muscles with contractures (with clinically short length) Studies are missing that investigated the influence of joint positions in case of bi-articular muscles during strength training and stretching Studies are missing that assessed the passive consequences of an increase in sarcomeres in series and in parallel	Focus on lengthening by addition of sarcomeres (both in serial and/or in parallel) Focus on influence of joint position on regional muscle stress and series of mobilization causing high global stress Focus on influence of sarcomeres in parallel and in series on passive muscle force and the force-length relationship
Humans		
Maturation	Lack of studies that examined natural muscle and tendinous tissue development Lack of studies reporting maturity status/biological age	Longitudinal studies in healthy subjects are needed Include a measure of biological age
Loading	Lack of studies that investigated the effects of concentric-only strength training on muscle belly length and physiological cross-sectional area Lack of studies that assessed the impact of loading strategies on microscopic muscle properties No information about the impact of connective tissue adaptations on muscle architectural changes Information is needed to clarify which components may evoke a dominant stimulus causing changes on muscle-tendon level after concentric, eccentric, and isometric training	Use of biopsies or *in-vivo* micro-endoscopy Focus on the effects on tendons and aponeuroses Investigate the relationship between muscle and tendinous tissue adaptations Focus on the following components: Contraction velocity Intensity Training muscle length Training joint range of motion (i.e., muscle excursion range)
Immobilization and stretching	Studies are missing that systematically examined the effects of short and long length immobilization on muscle-tendon properties Lack of studies on the effects of immobilization and stretching on microscopic muscle properties (e.g., number of sarcomeres in series and parallel)	Focus on the effects of long length immobilization on muscle growth and tendon stiffness Use of biopsies or *in-vivo* micro-endoscopy
Combined methods	Lack of studies that investigated the effects of static stretching combined with electrical stimulation on muscle-tendon properties Lack of knowledge about the effects of stretching on muscle growth when combined with strength training or strength exercises Only a few studies showing contrasting results of the effects of proprioceptive neuromuscular facilitation (PNF) stretching on muscle-tendon properties	Study the effects in healthy subjects Particularly focus on the effects of loaded interset-stretching Investigate the effects of PNF stretching regimen (duration >6weeks) on muscle-tendon properties

#### Muscle Development

In accordance with maturational processes in animals, human muscle growth is driven by bone elongation and related increases in body mass (Morse et al., 2008; Benard et al., 2011). Since human muscles already contain the full adult complement of muscle fibers by the time of birth (Carlson, 2019), existing muscle fibers increase in length and grow in diameter in proportion to the growth of the body. These growth mechanisms may be different from the onset of the pubertal age since sex hormones start to contribute, which results in a pronounced rise of whole-body muscle mass and strength during adolescence (Round et al., 1999). After adolescence, adult values are attained (van Praagh and Dore, 2002). These adaptations are also reflected in both increases in muscle CSA (Deighan et al., 2006) and muscle strength with age (Morse et al., 2008; Pitcher et al., 2012). Before puberty, increases in muscle force generating capacity during growth occur in parallel with increases in muscle CSA, whereas increases in strength exceed those in muscle CSA after puberty (see van Praagh and Dore, 2002 for summary).

Studies on gross muscle morphology of pennate muscles (knee extensors and plantar flexors) further reported smaller fascicle and muscle lengths, muscle volumes and PCSA in children when compared to adults (Morse et al., 2008; O’Brien et al., 2010b). Further investigations showed that increases in muscle volume likely result from an increase in fascicle length and PCSA (O’Brien et al., 2010b; Benard et al., 2011). Therefore, during childhood, an addition of sarcomeres in series and in parallel as reported for pennate animal muscles seems to occur. Besides, there is still no conclusive evidence about alterations of the fascicle PA with age (Binzoni et al., 2001; O’Brien et al., 2010b; Benard et al., 2011).

During pubescence, relatively higher gains in body mass than in body height and tibia length result in higher loads on the musculoskeletal system (Benard et al., 2011; Weide et al., 2015). Today, there is evidence that longitudinal growth of pennate muscles (e.g., GM muscle) during pubescence seems to be mediated by increase in PCSA solely (Weide et al., 2015) rather than by longitudinal fascicle growth, which is likely due to increased serum levels of growth factors (Rogol et al., 2002).

#### Tendon Development

Although neglected for decades, recent studies in humans demonstrated that tendons also adapt to biological maturation processes by alteration of their properties (Kubo et al., 2001b; O’Brien et al., 2010a; Waugh et al., 2012; Mogi et al., 2018). Age-related increases in body mass and force generating capabilities during maturation are associated with an increase in tendon loading that stimulates adaptations in tendinous tissue and changes the tendon’s mechanical properties (Waugh et al., 2012).

Several studies have shown that tendon length and CSA increase during childhood until adulthood (O’Brien et al., 2009; Waugh et al., 2012; Mogi et al., 2018). It seems that before the onset of the adolescent growth spurt (i.e., peak height velocity, PHV), longitudinal growth of the MTC results from increases in both the muscle belly and tendon lengths (O’Brien et al., 2010a; Mogi et al., 2018). After PHV, the longitudinal MTC growth results from increases in muscle length only (Mogi et al., 2018). Increases in tendon CSA mainly appear before the adolescent growth spurt (Mogi et al., 2018), whereby the CSA is highly variable and may even decrease around PHV (Neugebauer and Hawkins, 2012). Moreover, the relative Achilles tendon CSA (to body mass) was reported to be greater in (pre-) pubertal children than in adults (Kubo et al., 2014). Both findings indicate the occurrence of critical phases of tendon development during adolescence (see Mersmann et al., 2017 for review).

Tendons of pre-pubertal children seem to be less stiff when compared to those of adults, which was reported for both the Patellar tendon (O’Brien et al., 2010a) and tendinous structures (i.e., combined deep aponeurosis and distal tendon (Kubo et al., 2001b)). As reported for animals, tendon stiffness increases during childhood (Waugh et al., 2012). Consequently, the difference in tendon stiffness between children and adults disappears during adolescence (Kubo et al., 2001b). It could be shown that a significant increase in both Achilles tendon stiffness and Young’s modulus occurred at and/or around the PHV with no further changes thereafter (Mogi et al., 2018). Therefore, it is assumed that tendon stiffness does not increase linearly from child- to adulthood but predominantly around the PHV due to alterations in the material property since tendon CSA did not change during that growth phase. This finding may be supported by the observation that the inner tendon core is highly metabolically active until adulthood (Heinemeier et al., 2013; Gumucio et al., 2015). Adaptations in adult tendon may predominantly occur in its outmost layers, evoking tendon CSA increases (Gumucio et al., 2015).

### Adaptations of Muscle-Tendon Properties Due to Loading in Adults

Besides natural development, loading of the MTC also causes muscle-tendon adaptations in humans. However, to the best of our knowledge, it is still not clear which loading strategy may stimulate longitudinal muscle growth and may alter the MTC length and the range of active force exertion. Although the effects of pure concentric or eccentric contractions are difficult to evaluate in humans, several studies exist that focused on the effects of training interventions including only concentric, eccentric, or isometric contractions as stimuli. The effects are summarized in the following sections and the deduced research gaps are compiled in Table 1.

Although muscle growth may be enhanced by specific loading strategies already in adolescents (Lloyd and Oliver, 2012), studies on the effects in this population are scarce. Consequently, the main findings were based on studies in adults, mainly males in the age range of 20 to 30years and may only be partially transferred to young as well as elderly people. In this context it is also important to develop appropriate strategies considering the physical and psychological development of the children and adolescents (Lloyd and Oliver, 2012).

#### Concentric-Only Strength Training

There is evidence that concentric-only strength training (CT) leads to increases in muscle strength and enhanced muscle size as reported for several muscles (e.g., VL, quadriceps, biceps brachii) after 8–12weeks of training (Higbie et al., 1996; Housh et al., 1996; Seger et al., 1998; Farthing and Chilibeck, 2003; Moore et al., 2012; Franchi et al., 2014; Farup et al., 2014b; Quinlan et al., 2021). For instance, Franchi et al. (2014) found VL muscle volume increases of 8% after 10weeks of CT and increases in VL ACSA in the muscle’s mid and distal portions (11 and 2%, respectively). Farup et al. (2014b) further reported larger enhancements when the VL CSA was measured at mid-muscle-level. In contrast, no significant change in mean ACSA of the elbow flexors was observed after 12weeks of CT (Vikne et al., 2006). Interestingly, both Franchi et al. (2014) and Seger et al. (1998) found regional hypertrophy in the central region of the VL ACSA (11%) and total quadriceps ACSA (3.4%) after CT, respectively. Increases in quadriceps muscle volume were also observed by Quinlan et al. (2021) after only 4weeks of submaximal CT performed for 8weeks with a leg press in young (mean age: 23.3years) and older males (mean age: 69.1years).

Concerning changes in fascicle length and PA, only a few studies are available. Despite observed increases in VL fascicle lengths of 2% after 4weeks of CT (Franchi et al., 2015) and around ~6% after 10weeks of CT (Blazevich et al., 2007; Franchi et al., 2014), decreases in fascicle lengths of 11.8% and~11.7% were reported for the biceps femoris and supraspinatus after the performance of isokinetic CT (Kim et al., 2015; Timmins et al., 2016). Regarding changes in fascicle PA after CT, studies consistently showed increases in the VL (ranging from 13–30%), biceps femoris (21.1%) and supraspinatus (~22.7%; Blazevich et al., 2007; Franchi et al., 2014; Kim et al., 2015; Timmins et al., 2016; Quinlan et al., 2021). In this context, Quinlan et al. reported significant increases in VL PA already after 2weeks of submaximal CT in both young and older males. Note that the magnitude of the observed changes was higher in the younger compared to the older group.

Regarding muscle fiber hypertrophy, increases in VL type 1 fiber area of 12.5% were reported after 12weeks of isotonic CT that were even higher with protein supplementation (Farup et al., 2014a). VL type 2 fiber CSA increased by 25.7% exclusively in the subjects who received the protein supplementation (Farup et al., 2014a). In contrast, no change in VL fiber area was observed following a cycle ergometer training for 8weeks (LaStayo et al., 2000) and only slight non-significant increases were displayed after 12weeks of progressive isokinetic CT (Hortobagyi et al., 1996).

Seger et al. (1998) investigated the effects of CT on VL muscle fiber CSA of fibers type 1, 2A, and 2B: the absolute areas tended to increase (9.9–23.5%). Concerning the elbow flexors, no difference in fiber type 1 proportion in the biceps brachii was found after 12weeks of CT (Vikne et al., 2006). Despite that, a significant reduction in 2X fibers of 2.8% and a trend for reduction of the 2AX fibers was observed. Please note that both fiber types are subtypes of the fast twitch fibers. They are classified based on differential myosin heavy chain gene expression and hybrid myosin heavy chain gene expression (Talbot and Maves, 2016).

Only a few studies have yet examined the effects of CT on tendon properties and tendinous structures. After 12weeks of CT, Patellar tendon stiffness and Young’s modulus were reported to increase by 75.7% (Malliaras et al., 2013). Similarly, Patella tendon stiffness and Young’s modulus were already significiantly increased after 4weeks of CT in young as well as older males (Quinlan et al., 2021). Quinlan et al. further showed that while the observed changes in tendon mechanical properties stayed constant in the young males, they continued to increase from week 4 to week 8 in the older males suggesting a delayed response. Accordingly, a significant decrease was observed in tendon elongation and strain by Malliaras et al. (2013). In contrast, no change in Achilles tendon stiffness was reported after 6weeks of a heel-drop regimen (Morrissey et al., 2011). In the previous studies, the Patella tendon CSA and length remained unchanged (Malliaras et al., 2013; Quinlan et al., 2021), while increases in Patella tendon CSA (+14.9%) were found after a 12-week CT performed with whey protein supplementation (Farup et al., 2014b).

In summary, studies that investigated the effects of CT mainly showed its potential to stimulate muscle hypertrophy in both upper and lower limb muscles at variable rates and locations. However, no clear conclusion can be drawn about the underlying mechanisms. Franchi et al. (2014) suggested that CT leads to hypertrophy mainly through addition of sarcomeres in parallel. This idea might be supported by the findings of larger muscle PA observed after CT in several studies and evidence of enhanced fiber areas. Furthermore, CT also stimulates an increase in tendon stiffness likely due to changed tendon material properties. As discussed by Quinlan et al. (2021), muscle and tendon adaptations may occur simultaneously to maintain the efficacy of the whole MTC irrespective of the contraction mode since synchronous adaptations were also found after eccentric-only strength training (see subsequent section).

#### Eccentric-Only Strength Training

Similar to the effects of CT, eccentric-only strength training (ET) also leads to increased muscle strength and muscle hypertrophy. For instance, increases in muscle volumes were reported for the VL, total quadriceps muscle, and separate quadriceps muscles after 10weeks of isokinetic and overload ET (Higbie et al., 1996; Norrbrand et al., 2008; Franchi et al., 2014; Quinlan et al., 2021). Similarly, increases in muscle volume, ACSA, and PCSA of different hamstring muscles were found after performing the Nordic hamstring exercise (NHE; Bourne et al., 2017; Seymore et al., 2017) or a hip extension exercise (Bourne et al., 2017) for several weeks. Elbow flexor ACSA increased by 11% after 12weeks (Vikne et al., 2006) and biceps brachii muscle CSA by 6.5% after 9weeks of ET (Moore et al., 2012). Increases in muscle thickness were also reported for different muscles (Farthing and Chilibeck, 2003; Duclay et al., 2009; Baroni et al., 2013; Guilhem et al., 2013; Cadore et al., 2014; Franke et al., 2014; Leong et al., 2014; Kim et al., 2015; Timmins et al., 2016; Alonso-Fernandez et al., 2018). Interestingly, the comparison of the effects of ET performed with submaximal and supramaximal intensity (80 and 110% of the concentric 1RM, respectively) showed no different effect on biceps brachii thickness (Krentz et al., 2017). By comparing the effects of CT and ET on muscle ACSA, only Higbie et al. (1996) and Vikne et al. (2006) found a superior effect for the latter. Further information on the comparison can be found in a previous review (Franchi et al., 2017).

As for CT, a location-specific response in muscle hypertrophy after ET was reported (Seger et al., 1998; Franchi et al., 2014). However, after ET, significant increases in muscle ACSA occurred in the distal and not the central portion of the muscles (Seger et al., 1998; Franchi et al., 2014, respectively).

Significant increases in VL and rectus femoris fascicle lengths were already observed after 4weeks of ET (Baroni et al., 2013; Franchi et al., 2015; Quinlan et al., 2021) with similar increases in VL fascicle lengths (3.1%) observed after 10weeks of ET (Blazevich et al., 2007). Even higher increases (12–17.6%) were found after 10–12weeks of ET (Baroni et al., 2013; Franchi et al., 2014). Guilhem et al. (2013) reported no change in VL fascicle lengths after a 9-week isoload or isokinetic ET. In contrast, two volume-matched ET regimen (isokinetic vs. isotonic) performed for 6weeks resulted in longer fascicle lengths (14.4 and 14.7%, respectively) in another study (Coratella et al., 2015). Longer fascicle lengths were also reported for the GM (Duclay et al., 2009) and the biceps femoris (Potier et al., 2009; Timmins et al., 2016; Bourne et al., 2017; Alonso-Fernandez et al., 2018; Presland et al., 2018) after different ET interventions. Besides increases in fascicle lengths, no superior effect of high volume NHE training could be found compared to low NHE training (Presland et al., 2018). Contrastingly, no change in biceps femoris fascicle length was observed by Seymore et al. (2017) likely caused by training at shorter hamstring muscle length when compared to other studies. Similarly, supraspinatus fascicle length did not change after 8weeks of isokinetic ET (Kim et al., 2015). In summary, the mentioned studies support the idea that ET leads to longer fascicle lengths. However, please note that the results concerning biceps femoris fascicle length changes reported after some ET interventions should be considered with caution due to methodological issues regarding the extrapolation methods used to assess fascicle lengths (Franchi et al., 2020; Sarto et al., 2021), i.e., overestimations and underestimations of fascicle length indicating, among other things, a subject dependent accuracy of the methods (Franchi et al., 2020).

Concerning the PA, the reported changes after ET deliver no clear trend. Varying increases in VL fascicle PA (+3% to 21.4%), rectus femoris (+31%) and GM PA (+7.6%) have been reported after 4–10weeks of different ET interventions (isokinetic, isoload, cycling; Blazevich et al., 2007; Duclay et al., 2009; Guilhem et al., 2013; Franchi et al., 2014, 2015; Leong et al., 2014; Quinlan et al., 2021), whereas others observed no changes (Potier et al., 2009; Baroni et al., 2013; Coratella et al., 2015) or even a decrease in PA in different muscles (Timmins et al., 2016; Alonso-Fernandez et al., 2018; Presland et al., 2018).

In contrast to their findings on the effects of CT, several authors reported significant increases in VL fCSA after 8–12weeks of ET (Hortobagyi et al., 1996; LaStayo et al., 2000). Similarly, mean CSA increases of both type 1 and 2A fibers of the biceps brachii were also observed (Vikne et al., 2006). These findings indicate that eccentric loading may favor in particular the increase of type 2 fiber CSA (Franchi et al., 2017). In addition, independent of protein supplementation, similar increases in VL fiber type 1 area (on average+15.7%) were reported after ET when compared to CT (Farup et al., 2014a). In contrast, only Mayhew et al. (1995) observed a greater change in fiber type 2 CSA after CT compared to ET, and Seger et al. (1998) observed a decrease in VL fiber type 2A area (−14.9%) after 10weeks of eccentric isokinetic knee extensor training.

Guex et al. (2016) assessed the effects of 3-week isokinetic ET at slow velocity (30°/s) on biceps femoris muscle architecture and hamstring function when performed with the hamstrings at short and long muscle length. Although no group-by-time interactions could be reported, higher effect sizes were found for changes in the long muscle length training group. This study may imply that the training of range of motion or the muscle excursion range might be important for fascicle length adaptations. More details about the underlying mechanisms can be found in the subsequent section. In contrast, Sharifnezhad et al. (2014) did not find a superior effect of isokinetic ET performed at longer muscle length of the VL or, in agreement with Krentz et al. (2017), higher intensity of the training after 10weeks (Sharifnezhad et al., 2014). However, the authors reported an increase in fascicle length of 14% after ET only when performed with a high (240°/s) but not a low fascicle lengthening velocity (90°/s).

Furthermore, there is evidence that ET leads to increases in tendon stiffness (Duclay et al., 2009; Malliaras et al., 2013; Geremia et al., 2018; Quinlan et al., 2021) and Young’s modulus (Malliaras et al., 2013; Geremia et al., 2018; Quinlan et al., 2021). In contrast, some studies have reported a lack of significant change (Mahieu et al., 2008) or even a decrease in stiffness (Morrissey et al., 2011). Since loading intensity seems to be key for tendon adaptation (e.g., Arampatzis et al., 2007, 2010), the differences between studies might be related to the applied loads or the duration of the training programs (Bohm et al., 2015). However, Quinlan et al. (2021) did not find a difference in neither absolute nor percentage change in tendon stiffness and Young’s modulus between ET and CT at any training duration (after 2, 4, 6, and 8weeks of training) or between age groups (younger vs. older adults). Their findings support the idea that the direction of strain applied to a tendon may not be decisive to evoke changes in its properties. Finally, increases in tendon CSA (+9.7%) were reported after 12weeks of ET (Farup et al., 2014b) with even higher increases (+15%) observed after 8weeks of high-load ET (Geremia et al., 2018).

#### Isometric Strength Training

Since isometric contractions allow for a controlled application of force within pain-free joint angles (Oranchuk et al., 2019), isometric strength training (IT) is often used in clinical settings. Therefore, it is important to know whether relevant adaptations in muscle-tendon properties occur.

Recent studies provided evidence that also IT leads to significant muscle hypertrophy. For instance, increases in muscle volume were reported for the quadriceps (Kubo et al., 2006b; Noorkõiv et al., 2014; Balshaw et al., 2016). Increases in VL muscle thickness were observed after 8weeks of IT depending on muscle site and training muscle length (Alegre et al., 2014) and after 14weeks of IT (~16.5%) performed with varying periodization (Ullrich et al., 2015). Similarly, increased volume of the triceps brachii (+12.4%) was reported after 10weeks of high-intensity IT (Kanehisa et al., 2002). Noteworthy, explosively performed IT did not result in significant changes in quadriceps muscle volume (Balshaw et al., 2016).

Two studies (Alegre et al., 2014; Noorkõiv et al., 2014) indicated a superior effect of IT performed at long muscle length (LML) to enhance VL muscle volume and thickness in comparison to training at short muscle length (SML). A shift of peak torque production to the training muscle lengths was also observed (Alegre et al., 2014), with a greater change found in the LML training group. As discussed by Alegre et al. (2014), the mechanisms underlying superior hypertrophic effects of training at LML could be influenced by several factors. For instance, IT performed at LML may have caused greater damage, therefore, greater adaptation by placing the muscle fascicles under greater stretch in comparison to training at SML. Furthermore, smaller moment arms resulting from greater flexed knee angles may induce greater mechanical fascicle stress. Noorkõiv et al. (2014) further highlighted the role of upregulation of mechanosensitive signaling mechanisms and augmentation of calcium signaling cascades that may only become critical when the target muscle was activated at long length. Finally, training-dependent releases of insulin-like growth factor or mechano growth factors may also be related to training muscle length. Furthermore, Oranchuk et al. (2019) deduced that training intensity seems to have a small effect on the hypertrophic responses, though it may have to reach a certain threshold (>20% of maximum voluntary contraction). In general, higher training volume had a superior effect on the hypertrophic responses when IT is performed.

Information about the effects of IT on muscle fascicle lengths and PA is scarce and inconsistent. Increases in VL fascicle length have been reported at the mid portion of the femur (+5.6%) after IT at SML, whereas increases in the distal portion (+5.8%) were observed after training at LML (Noorkõiv et al., 2014). This result may indicate specific adaptations in fascicle lengths regarding training muscle length chosen during IT. Furthermore, increases in VL fascicle lengths of ~16.5% have been observed after IT conducted with the knee joint at 70° (Ullrich et al., 2015), which lies between the angles chosen in the study of Noorkõiv et al. In contrast, Alegre et al. (2014) did not find changes in fascicle lengths after IT at LML and other muscles than the VL. Significant increases (+11.7%) in VL PA were only found after IT at LML (Noorkõiv et al., 2014) and in the long head of the triceps brachii (~15.5%) after high- and low-intensity IT (Kanehisa et al., 2002). No change in PA was observed after training at SML (Noorkõiv et al., 2014) and in the study of Ullrich et al. (2015).

Increases in VL tendon-aponeurosis stiffness were observed after 12weeks of IT at LML (Kubo et al., 2006b; Massey et al., 2018) with no significant change after training at SML (Kubo et al., 2006b). It was assumed that the higher mechanical stress applied in the LML training (due to shorter moment arm length) might have caused the differences between groups. Increased Patella tendon stiffness (+20% and+16%) and Young’s modulus (+22% and+16%) were observed after explosive IT and IT, respectively, with no significant differences between the interventions (Massey et al., 2018). VL aponeurosis hypertrophy (+7%), assumed to provide an enlarged attachment area for increased muscle CSA, was only found after non-explosively performed IT, which was in line with the greater volume observed in this study. In agreement, increases in Achilles tendon-aponeurosis stiffness (+36.0%), elastic modulus (~18.2%) and region-specific hypertrophy were found after 14weeks of high-strain, but not low-strain IT (Arampatzis et al., 2007), supporting the results of previous studies (Kubo et al., 2001a, 2006a). Arampatzis et al. (2010) also showed that a higher tendon strain duration per contraction may evoke superior adaptational responses. No change in Achilles tendon CSA, but increases in tendon-aponeurosis stiffness and Young’s modulus were also found after 12weeks of both isokinetic IT performed with short and long rest intervals between isometric repetitions performed at high-strain (Waugh et al., 2018). However, tendons trained with IT and shorter rest duration led to a reduction in collagen organization in the same study.

### Unloading Strategies, Immobilization and Stretching

In contrast to animal studies, human immobilization studies have mainly focused on pennate muscles. There is consistent evidence that immobilization in humans results in decreased muscle strength and size alongside negative changes in peripheral and central neuromuscular function (Campbell et al., 2019). The extent of adaptations might be related to the ground-based model (e.g., bed rest, limb casting) used (Campbell et al., 2019). Furthermore, it appears that the time course of the adaptations varies with prolonged unloading: the greatest rate of change in muscle strength may occur in the first weeks (approx. in the first two weeks (de Boer et al., 2007)), prior to significant changes in muscle structure (Seynnes et al., 2008b; Campbell et al., 2019). Faster deterioration in muscle function might be explained by changes in neural processes (i.e., generation and transmission of neural activation signals, transmission to and action of the contractile apparatus; Campbell et al., 2019).

Most of the mentioned findings are based on studies in which the target muscles were immobilized in a short position (e.g., with the knee joint in extended position or the ankle joint kept in plantarflexed position as in bed rest studies). In those studies, negative effects of short length immobilization on gross muscle morphology and microscopic muscle properties such as fCSA have been reported (e.g., Hortobagyi et al., 2000; Kubo et al., 2000, 2004; Kawakami et al., 2001; de Boer et al., 2007; Suetta et al., 2009; Hvid et al., 2010, 2013; Oates et al., 2010; Belavy et al., 2017). However, to receive a clear picture about how immobilization affects muscle length growth, we further summarized studies’ findings on the effects of long length immobilization.

#### Effects of Long Length Immobilization on Muscle Properties

To gain information about the effects of long length immobilization, studies that reported immobilization of the knee joint in either 60 or 70 degrees of flexion and/or ankle joint angles in 90 degrees flexion were summarized.

In accordance with the results of short length immobilization, reductions in isometric force of −13% up to −28% after 7days (Deschenes et al., 2009, 2012) and~−25.5% after 14days (Mitchell et al., 2018) of unilateral lower limb suspension (ULLS) immobilization could be found for the knee extensors. The isometric force of the plantar flexors declined by −10% after 14days of a similar ankle joint immobilization (Seynnes et al., 2008b). Furthermore, decreases in isokinetic strength and muscle performance variables (e.g., total work) were reported (Deschenes et al., 2009).

Plantar flexor muscle volumes decreased after 2weeks of ULLS (~−5.3%) with higher decreases (−7.7%) present after 23days (Seynnes et al., 2008b). Furthermore, reductions of both GL fascicle length and fascicle PA were observed, which became significant after 23days (−4% and−5%, respectively). This was accompanied by declines (−3%) of GL PCSA after 14days, which did not further decrease until 23days due to a faster decrease in fascicle length then muscle volume (Seynnes et al., 2008b). Similar to short length immobilization, immobilization at long lengths resulted in decreased PCSA of the VL, rectus femoris, and total quadriceps in men and women (~−6.2%, ~−3.3% and~−5.8%, respectively) after 2weeks of knee bracing (Yasuda et al., 2005). Declines in fCSA were also observed, with similar reductions reported by Oates et al. (2010). These declines seem to be smaller than those observed after 2weeks of short length immobilization of the knee extensors (Hvid et al., 2010). However, similar values were also reported (Hortobagyi et al., 2000).

In conclusion, long length immobilization seems to result in reductions of sarcomeres in series and in parallel in humans. Future studies are needed for clarification.

#### Effects of Immobilization on Tendon Properties

Concerning the effects of immobilization on tendon properties, it is assumed that joint positioning and therefore tendon length may play a role for the amount of deterioration with disuse (Maganaris et al., 2006). Tendons may be more vulnerable to material and structural deterioration at shorter than longer lengths (Maganaris et al., 2006), however, to the best of our knowledge, this has not yet been verified. Since information on the joint position was not always retrievable from the studies, this assumption could neither be verified nor falsified in this review.

Studies showed that both short-term (2–3weeks, e.g., Kubo et al., 2004; Reeves et al., 2005; de Boer et al., 2007; Shin et al., 2008; Seynnes et al., 2008a; Couppe et al., 2012) as well as long-term (12weeks, Reeves et al., 2005) immobilization may affect the tendon mechanical and material properties. Patellar tendon stiffness decreases of 9.8 to 19.7% after 2 (de Boer et al., 2007; Couppe et al., 2012), ~30.7% after ~3 (Kubo et al., 2000; de Boer et al., 2007) and 58% after ~12weeks (Reeves et al., 2005) of immobilization have been reported. Comparing the results regarding differences in knee joint immobilization angle (10 degrees vs. 30 degrees by de Boer et al., 2007; Couppe et al., 2012, respectively), it could be suggested that immobilization at shorter patellar tendon length led to higher declines in tendon stiffness. However, due to the high variability between studies (e.g., duration of immobilization), further results cannot support this hypothesis. Nevertheless, the results show that disuse mainly affects the material properties rather than tendon CSA (de Boer et al., 2007; Shin et al., 2008; Kinugasa et al., 2010; Couppe et al., 2012). Most studies (except for Kinugasa et al., 2010) reported unchanged tendon length and CSA after short-term (Kubo et al., 2004; de Boer et al., 2007; Shin et al., 2008; Couppe et al., 2012) and prolonged unloading (Reeves et al., 2005). It is assumed that the material changes might be caused by adaptations in the collagen fibers (see Kinugasa et al., 2010 for summary) as reported in animal studies (Vailas et al., 1988; Nakagawa et al., 1989). The resulting changes in tendon stiffness may negatively affect, for example, force transmission and the electromechanical delay (Kubo et al., 2000; Reeves et al., 2005; de Boer et al., 2007) since the muscle has to shorten further to stretch the tendon (Maganaris et al., 2006).

Based on the findings of Maganaris et al. (2006) who investigated the effects of chronic unloading due to paralysis and found reductions of patellar tendon CSA of 17%, it can be assumed that changes in the tendon morphology may only occur after years of disuse (lesion duration: 1.5 to 24years).

#### Passive Static Stretching

Stretching is often used in sports for injury prevention and to speed up the recovery of the athletes. In clinical practice it is a common treatment approach to counteract, for instance, muscle contractures. Despite evidence that prolonged stretching for several weeks can improve joint range of motion (e.g., Blazevich et al., 2014), only trivial effects for reductions in muscle stiffness were reported (Freitas et al., 2018). Furthermore, it is still not clear if this approach can evoke muscle growth.

Only recently, Nunes et al. (2020) reviewed current studies on the effects of static stretching with the aim to elucidate whether stretch training is capable to elicit muscle hypertrophy in humans. Out of 10 studies only 3 reported positive effects in specific muscle size parameters (Nunes et al., 2020). Mizuno (2017) investigated the effects of 8-week static stretching only and static stretching combined with electrical stimulation (see section 4.4.1) on GM muscle architectural parameters. In contrast to the control group, GM muscle thickness was significantly increased (+5.8%) in the static stretching group (*n*=12), whereas no change in PA could be found (Mizuno, 2017). Increases in gastrocnemii muscle thickness were also found by Simpson et al. (2017) after 6weeks of loaded stretch training. However, this result was questioned by other authors (Jakobi et al., 2018; Nunes et al., 2018). Additionally, increases in muscle fascicle lengths, with differing amounts according to measurement site (distal or central region of the muscle), muscle (GM vs. GL), and time period (e.g., baseline, day 5, week 6), were also reported (Simpson et al., 2017). Decreases in PA were found in the GL muscle at both sites at week 6 and 1-week post-training, whereas the PA was increased (+4.9%) in the GM muscle 1-week post-training when measured distally. Increases in biceps femoris fascicle lengths of 13.6% were found after 8weeks of high-intensity stretching training in the pilot study of Freitas and Mil-Homens (2015). The training was performed 5 times a week with continuous stretching for 450s, in which torque was increased every 90s until discomfort was reported. Decreases in PA (−15.1%) did not reach statistical significance. Despite a small sample size, this finding implies a potential of static stretching to induce longitudinal muscle growth if applied for a longer time period with a high intensity (i.e., non-rest protocol; Freitas and Mil-Homens, 2015). This is supported by a cross-sectional study of Moltubakk et al. (2018) who showed that ballet dancers, accustomed to regular stretching training over years, have longer gastrocnemius fascicle and Achilles tendon lengths compared to healthy controls. Sarcomerogenesis due to chronical stretch stimulus in VL fascicles of a 16-year-old girl following 8month of surgical femur lengthening was also shown by Boakes et al. (2007).

Furthermore, the idea that stretching intensity (i.e., high strain placed upon the muscle) might be a key factor to evoke adaptations on muscular level is also supported by findings of recent studies (e.g., Longo et al., 2021; Moltubakk et al., 2021; Panidi et al., 2021; Yahata et al., 2021). In contrast to Freitas and Mil-Homens (2015) and Panidi et al. (2021), Yahata et al. and Moltubakk et al. did not find changes in muscle architectural properties (i.e., fascicle lengths) when static stretching was performed either with a high stretching volume (6 sets of 5min/day, 2days/week, 5weeks, in total 18.000s) or for a longer period of time (24weeks; in total 40.320s of static stretching), respectively. As concluded by Yahata et al. (2021), very high volumes of static stretching training may not substitute muscle strain applied with low intensity.

We finally emphasize that the studies which reported hypertrophic effects all used an apparatus that may have ensured a high stretching (over-) load (Nunes et al., 2020). Therefore, using an apparatus/machine might be advantageous when compared to self-applied stretching by inducing a higher stimulus/intensity. However, also other factors, e.g., the total amount of or rest duration between stretches, may be important.

Studies that have investigated the effects of long-term static stretching on tendon properties are scarce, with limited evidence supporting no changes in tendinous tissues, at least when performed up to 6weeks. In this context, 30 sessions of overloaded stretch training did not alter Achilles tendon length or tendon thickness (Simpson et al., 2017). Additionally, Konrad and Tilp (2014) reported no change in passive Achilles tendon stiffness after 6weeks of plantarflexor stretching (Konrad and Tilp, 2014). In agreement, Kubo et al. (2002a) investigated the effects of 3-week static stretching (two times a day, five stretches for 45s, 15s rest between stretches) on the viscoelastic properties of human tendon structures (i.e., stiffness and hysteresis). The stretching training did not result in changes in stiffness but in significantly decreased hysteresis. Based on this finding, the authors concluded that static stretching does not affect the serial elastic component but may affect the connective tissue elements in parallel with the muscle fibers (i.e., the endomysium, perimysium, and epimysium).

### Combined Methods—Stretching and Loading

#### Static Stretching and Electrical Stimulation

Mizuno (2017) also investigated if static stretching combined with electrical stimulation might be superior in altering the GM muscle architectural properties when compared to static stretching alone. In the combined group, the muscle was contracted during the stretches as well as during the resting period (Mizuno, 2017). The authors reported a similar increase in GM muscle thickness for the static stretching and static stretching/stimulation group, therefore demonstrating no increased benefit of the combined approach.

#### Stretching and Strengthening

There are a few studies that have investigated the effects of stretching on muscle hypertrophy when combined with strength training or strength exercises (e.g., Kubo et al., 2002b; Junior et al., 2017; Ferreira-Júnior et al., 2019). A brief overview of the results can be found in Nunes et al. (2020). The studies either showed reductions or no impairment of the hypertrophic responses when stretching was performed immediately before (Junior et al., 2017; Ferreira-Júnior et al., 2019) or separated from the strength training sessions (Kubo et al., 2002b). Still, no clear conclusions can be drawn.

Interestingly, it appears that loaded interset-stretching, i.e., stretching performed between the sets of strength exercises, may have a positive additional effect on strength and extensibility (Souza et al., 2013), and the occurring hypertrophic responses (Silva et al., 2014; Evangelista et al., 2019). For instance, Silva et al. (2014) reported greater increases in GM muscle thickness in the interest- stretching group after calf strengthening for 5weeks. Additionally, Evangelista et al. (2019) found greater changes in VL thickness and the summed thickness of 4 muscles when compared to the results of a traditional strength training program. Future work has to verify the additional benefit of interest stretches on hypertrophic responses.

#### Proprioceptive Neuromuscular Facilitation Stretching

Proprioceptive neuromuscular facilitation (PNF) stretching is commonly used with the aim to increase the joint range of motion and to lengthen the MTC (Sharman et al., 2006). Compared to static stretching, PNF further involves a static isometric (maximal) contraction of the stretched target muscle and/or a concentric contraction of the opposing muscle in order to lengthen the target muscle applied during the stretching procedure (Sharman et al., 2006).

Konrad et al. (2015) investigated the effects of a 6-week PNF stretching performed 5 times a week on muscle and tendon stiffness as well as GM muscle architecture and observed a slightly enhanced PA but no changes in muscle fascicle lengths after the intervention (Konrad et al., 2015). Furthermore, no changes in MTC and GM muscle stiffness appeared, whereas decreases in Achilles tendon stiffness were observed. In contrast, Mahieu et al. (2009) demonstrated no alterations in Achilles tendon stiffness after 6weeks of PNF stretching (Mahieu et al., 2009).

In summary, no clear statement about the effects of PNF stretching on muscle growth can be deduced yet.

## Conclusions and Clinical Implications

The main aim of this review was to identify potentially efficient strategies to induce longitudinal muscle growth in humans. The knowledge on muscle and tendon growth and adaptation provides important implications for treatments that aim to interact with the MTC. Therefore, we summarized studies on natural muscle growth and reviewed different treatments and training strategies. Finally, we also highlighted potentially important research gaps (Table 1).

### Typical Growth in Humans

There is evidence that addition of sarcomeres in series and increases in fCSA are the important contributors for muscle belly growth in children. Longitudinal and radial growth of tendon and aponeurosis contribute to the length range and extensibility of the MTC. Animal and human studies showed that tendons are highly compliant and adaptable at young age, which might influence treatment outcomes (section “Effects of Immobilization on Muscle Growth”). During pubescence, length growth of pennate muscles seems to be mediated by increases in PCSA alone, likely due to growth factors. In adulthood, muscle growth, i.e., adaptation, seems to be dependent on the loading applied to the muscles.

### Effects of Strength Training on Muscle Growth

In contrast to CT that may induce longitudinal muscle growth by increases in fCSA, ET seems to trigger the addition of serial-sarcomeres as indicated by longer muscle fascicle lengths. These mechanisms of architectural remodeling seem further not to be impacted by age. However, research findings are variable and the underlying mechanisms have still to be clarified. Further information on muscle remodeling due to CT compared to ET can be found by Franchi et al. (2017). Since IT resulted in muscle hypertrophy and may also increase muscle fascicle lengths, it might be an appropriate training regimen especially for clinical populations as it allows controlled supervision of force within pain-free joint angles. From animal studies it appears that the muscle length at which the training is performed might be decisive for the efficiency with a tendency to greater changes after training at long muscle length. Furthermore, training intensity might also play an important role. Nevertheless, more studies have to be performed to elucidate the underlying mechanisms of the observed adaptations and the role of the chosen muscle length.

Changes of training regimen regarding these aspects can be easily made with regard to healthy people and athletes. However, when it comes to clinical populations, there will be barriers restricting the implementation. Despite the fact that strength training may not be easy to perform with specific patient populations at all, focusing on muscle length and sufficient muscle excursion as well as applying a high intensity might be additionally problematic, for instance, due to altered muscle-morphometrics and reduced patient compliance, respectively. Although IT allows controlled supervision of force within pain-free joint angles and may evoke longitudinal muscle growth, limited selective motor control may restrict the desired adaptations. In this case, electrical stimulation could be of help, but is often not tolerated, especially by younger patients. Similarly, eccentric and concentric strength training are not easy to perform and specific (individual) approaches and devices supporting/guiding the required movements are needed.

All three strength training strategies resulted in changes of tendon properties. However, it is still not clear how, for instance, increased tendon stiffness may influence muscle morphological properties and therefore longitudinal muscle growth (see also section The effects of stretching on muscle growth).

### Effects of Immobilization on Muscle Growth

Evidence exists that immobilization of pennate muscles at long muscle length in animals does not result in an addition of sarcomeres in series similar to parallel fibered muscles. Furthermore, in young animals, immobilization of the soleus muscle resulted in a lower serial-sarcomere number and increased tendon length, possibly as adaptation to decrease the tension. In humans, immobilization of pennate muscles resulted in muscle atrophy (e.g., decreases in fCSA, PCSA) independent of the chosen muscle position (short or long length). Additionally, stiffness’ decreases and changes in the material properties of tendons were also observed that may negatively affect the interaction between muscles and tendons. These findings are important since commonly used treatments such as serial casting or orthotics are based on the theory that a muscle adapts to an imposed lengthening stimulus as shown in animal studies. At least in children with CP, recent casting and orthotic studies already demonstrated the mentioned (negative) adaptations in muscle and tendinous tissues (McNee et al., 2006; Hösl et al., 2015; Peeters et al., 2018).

### The Effects of Stretching on Muscle Growth

Based on the animal studies, it was further assumed that traditional stretching treatments such as static stretching also induce longitudinal muscle growth in humans (e.g., Wiart et al., 2008; Zhao et al., 2011). With regard to static stretching in healthy populations, there is slight evidence that high-intensity or a high stretching (over-) load over a longer period of time (> 8weeks) is needed to induce longitudinal muscle growth (Freitas and Mil-Homens, 2015). Based on animal studies that reported increases in muscle length, the approach to combine static stretching with electrical stimulation or strength exercises might be worth to be investigated, especially in clinical populations. However, to date, studies supporting an increased benefit of such an approach in humans are still lacking. Similarly, the effects and the potential of loaded inter-set stretching during a strength training regimen should be assessed in future studies since such approaches could be easily integrated in the training routines of athletes.

Besides a lack of studies that have evaluated the impact of PNF stretching on muscle-tendon properties in healthy subjects, hold-relax PNF stretching performed with a robotic ankle-foot rehabilitation system in hemiplegic poststroke patients resulted in decreased tendon length (Zhou et al., 2016). Consequently, investigating the effects of PNF on tendon properties, especially, changes in tendon stiffness, and possibly related adaptations in muscle properties could be a promising research direction.

### Influences of Intermuscular and Adjacent Connective Tissue on (Regional) Muscle Adaptation

The addition of sarcomeres in series as an adaptive effect is hypothesized to occur as a regulatory effect to assure that each single sarcomere is at its optimum length on the active length-force curve during common activities (Herring et al., 1984). However, stress caused by acutely lenghtening the muscle is not uniform throughout the muscle (e.g., Yucesoy et al., 2003). This latter explains local addition of sarcomeres, such that in some muscle regions additions may occure as an effect to the lenghtening intervention, whereas in other regions this might be absent. Further research concerning the regional muscle adaptation caused by strength training, immobilization and stretching is needed.

*In vivo*, muscle force is transmitted through the myotendinous pathway, but also through intra- and extra-muscular connective tissue (Loeb, 1984; Trotter, 1990; Trotter et al., 1995; Huijing and Baan, 2001; Maas et al., 2001; Huijing, 2002; Yucesoy et al., 2003). This latter consists of intermuscular and adjacent connective tissue. The mechanical interaction between adjacent muscles and connective tissue is an important factor that has to be considered when performing lengthening interventions. The presence of mechanical interaction between adjacent muscles generates asymmetrical strain on target muscles when lengthened. Besides, exerted forces are dependent on the relative position of adjacent muscles (Maas et al., 2001, 2003, 2004). Further research concerning the influence of intermuscular and adjacent connective tissue on muscle adaptation during muscle lengthening interventions is needed.

## Final Conclusion

In conclusion, strategies that positively affect longitudinal muscle growth include activities at long muscle lengths (either during a stretching maneuver, immobilization, or active contraction) at high intensities for a longer period of time (> 8weeks). While concentric and isometric strength training potentially increase muscle length due to increased fCSA in pennate muscles, eccentric strength training seems to increase fascicle length and therefore may result in longitudinal muscle growth. However, with regard to the different strategies and their effects on muscle-tendon properties, several research gaps exist that have to be filled in order to find the most efficient strategy.

## Author Contributions

AK and RJ developed the idea of the current manuscript. AK and CR performed the literature searches, summarized the studies’ results, and prepared the current manuscript. All authors contributed to both the interpretation and discussion of the results and critically revised and edited the manuscript. All authors read and approved the submitted version.

## Funding

This work was supported by the Austrian Science Fund (grant number T 1017).

## Conflict of Interest

The authors declare that the research was conducted in the absence of any commercial or financial relationships that could be construed as a potential conflict of interest.

## Publisher’s Note

All claims expressed in this article are solely those of the authors and do not necessarily represent those of their affiliated organizations, or those of the publisher, the editors and the reviewers. Any product that may be evaluated in this article, or claim that may be made by its manufacturer, is not guaranteed or endorsed by the publisher.
